# Snoopy’s hybrid simulator: a tool to construct and simulate hybrid biological models

**DOI:** 10.1186/s12918-017-0449-6

**Published:** 2017-07-28

**Authors:** Mostafa Herajy, Fei Liu, Christian Rohr, Monika Heiner

**Affiliations:** 1 Department of Mathematics and Computer Science, Faculty of Science, Port Said University, Port Said, 42521 Egypt; 2 School of Software Engineering, South China University of Technology, Guangzhou, 510006 People’s Republic of China; 3 Computer Science Institute, Brandenburg University of Technology, Cottbus, 10 13 44 Germany

**Keywords:** Hybrid simulation, Hybrid Petri nets, Hybrid biological models, Snoopy

## Abstract

**Background:**

Hybrid simulation of (computational) biochemical reaction networks, which combines stochastic and deterministic dynamics, is an important direction to tackle future challenges due to complex and multi-scale models. Inherently hybrid computational models of biochemical networks entail two time scales: fast and slow. Therefore, it is intricate to efficiently and accurately analyse them using only either deterministic or stochastic simulation. However, there are only a few software tools that support such an approach. These tools are often limited with respect to the number as well as the functionalities of the provided hybrid simulation algorithms.

**Results:**

We present Snoopy’s hybrid simulator, an efficient hybrid simulation software which builds on Snoopy, a tool to construct and simulate Petri nets. Snoopy’s hybrid simulator provides a wide range of state-of-the-art hybrid simulation algorithms. Using this tool, a computational model of biochemical networks can be constructed using a (coloured) hybrid Petri net’s graphical notations, or imported from other compatible formats (e.g. SBML), and afterwards executed via dynamic or static hybrid simulation.

**Conclusion:**

Snoopy’s hybrid simulator is a platform-independent tool providing an accurate and efficient simulation of hybrid (biological) models. It can be downloaded free of charge as part of Snoopy from http://www-dssz.informatik.tu-cottbus.de/DSSZ/Software/Snoopy.

**Electronic supplementary material:**

The online version of this article (doi:10.1186/s12918-017-0449-6) contains supplementary material, which is available to authorized users.

## Background

In order to study the dynamics of biological models, a simulation procedure is usually employed to emulate reaction firings. A vector representing the state of a system serves to track the species concentrations and/or the corresponding number of molecules as the simulation advances with respect to time. The chosen simulation procedure determines how the system state vector is updated as well as the progression of the simulation time. There are various approaches to capture reaction firings as well as their effects on the system state. However, all available algorithms can be grouped into four categories: stochastic, approximate stochastic, deterministic, and hybrid simulation approaches [1, 2].

Stochastic simulation methods [2–4] consider reaction firings as a random process and each reaction is executed individually. Therefore, stochastic simulation is very accurate compared to approximate approaches (e.g., approximate stochastic methods and deterministic ones). However stochastic simulation algorithms (SSA) are often referred to as computationally inefficient as they may consume much runtime to accomplish the discrete and individual firing of reactions. They can be used to simulate models with a moderate amount of reactions that do not fire too frequently, since, increasing the number of reactions could at the same time increase the number of stochastic events. As an improvement of the exact stochastic simulation, approximate stochastic simulation algorithms [5] group and fire multiple reactions at every step. Thus, they can save considerable runtime. Nevertheless, they will still require rather expensive computations.

On the contrary, deterministic simulation offers a completely different approach by considering reaction firing as a deterministic process which approximates reaction firings by constructing a system of ordinary differential equations (ODEs) or by using other approximation techniques (e.g., see [6]). Although deterministic simulation is computationally efficient, the results are not accurate for all kinds of computational models of biochemical reaction networks [2]. For instance, deterministic simulation is not applicable for many experiments, where molecular fluctuations of species with a few number of molecules drive the overall model behaviour (for examples see [7, 8]).

As a combined approach, hybrid simulation [9–15] merges exact stochastic and approximate algorithms. Thus, it takes advantage of computational efficiency, while avoiding result inaccuracy. Hybrid simulation works by first partitioning the set of reactions into stochastic and deterministic ones and correspondingly classifying the set of species into discrete and continuous ones. Afterwards, a system of ODEs is constructed for the deterministic regime using kinetic rate laws as specified (e.g., mass action). The system of ODEs is numerically integrated until a stochastic reaction is to occur and then the stochastic reaction takes place. The whole procedure is repeated until the end of the simulation time is reached.

However, the implementation of hybrid simulation is not a straightforward task compared with the comparably simple stochastic simulation methods, since it requires the interplay and integration of an ODE solver in addition to the SSA. Hence, it becomes intricate to write a dedicated and efficient simulation code for each model. Therefore efficient hybrid simulation software tools are required to accelerate the model development and execution. Unlike stochastic simulation, there are only a few software tools that currently support hybrid simulation (see e.g., [16, 17]). Furthermore, the original hybrid simulation algorithm introduced in [9] is not efficient to simulate all kinds of models. For example, a high frequency of reaction events leads to a performance drop. Therefore, recent hybrid approaches employ more sophisticated techniques in order to achieve a better performance (see e.g., [6, 12, 13]). Besides, hybrid simulation tools should continuously evolve and support the state of the art of hybrid simulation approaches such that they can cope with the continuously growing interest in systems biology.

In this paper, we present Snoopy’s hybrid simulator, an efficient and generic (i.e., it does not assume a special kind of biochemical network models) hybrid simulator that supports state-of-the-art hybrid simulation approaches. Snoopy’s hybrid simulator is deployed as a component of the Petri net tool Snoopy [18] and its steering server [19]. The latter tool permits different simulation scenarios than the one discussed in this paper (please see [20] for more details). Snoopy’s hybrid simulator has been recently restructured to support recent advances in hybrid simulation algorithms. Moreover, it admits a graphical representation of biochemical reactions by means of Petri nets (see below), while complex models that exhibit repeated components can be easily constructed as coloured Petri nets [15]. Snoopy’s hybrid simulator is a free software tool that can run on many well-known platforms including MS Window, MacOSX and some Linux distributions. A comprehensive user manual is available at [21].

**Modelling biochemical networks via Petri nets.** Petri nets, as a discrete modelling approach, have been widely applied in many fields, including systems biology [22, 23]. In Petri nets, tokens on places represent discrete quantities of species such as the number of molecules or levels of species concentration. To accommodate different modelling scenarios, Petri nets have been extended in many ways [23]. For instance, stochastic Petri nets ($\mathcal {SPN}$) [22] were proposed by associating each transition with an exponentially distributed waiting time, and continuous Petri nets ($\mathcal {CPN}$) have been introduced to support continuous markings (cf., [22, 24]). The underlying semantics of a ${\mathcal {CPN}}$ model is a system of ODEs. However, there are different ${\mathcal {CPN}}$ interpretations. We adopt a special semantics of ${\mathcal {CPN}}$ called bio-semantics (cf., [25]). In the bio-semantics, we assume that transition rate equations are defined in terms of kinetic rate laws (e.g., mass action) that are commonly used to model biochemical networks. This assumption will considerably simplify the ${\mathcal {CPN}}$ simulation and its implementation for this particular application.

Furthermore, in order to allow discrete and continuous entities to coexist in one model, different types of hybrid Petri nets (${\mathcal {HPN}}$) were proposed for different purposes [24]. We employ a special class of ${\mathcal {HPN}}$ called generalised hybrid Petri nets (${\mathcal {GHPN}}$) [11] which is specifically tailored to the modelling of biochemical reaction networks. ${\mathcal {GHPN}}$ offer two types of places and five types of transitions, which permit together the convenient modelling of various kinds of (biological) processes. A detailed description of ${\mathcal {GHPN}}$ can be found in [11, 21].

**Fig. 1:**
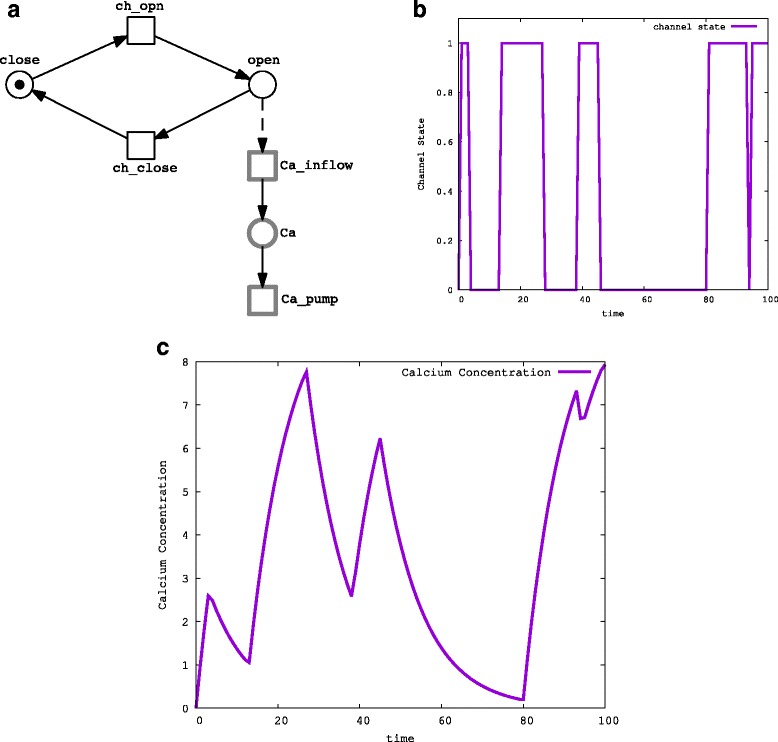
A simple example illustrating the operation of hybrid Petri nets: (**a**) the HPN representation of simplified calcium dynamics, (**b**) a time course of opening and closing of the calcium channel, and (**c**) the corresponding calcium concentration. The two discrete places *close* and *open* represent the channel states, closed and opened, respectively. The two stochastic transitions: *ch_open* and *ch_close* model the state transition of the channel. The continuous place *Ca* models the calcium concentration. The calcium inflow is modelled via the continuous transition *Ca_inflow* which has a rate proportional to the open state of the channel. The outflow of the calcium is modelled using the transition *Ca_pump*. The simulation result of the model is given in (**b**) and (**c**)

Figure 1 presents an introductory example of using ${\mathcal {GHPN}}$ to model biochemical reaction networks. We follow a simplified scenario of the calcium dynamics detailed in [26]. Intracellular calcium dynamics is a complex process which requires hybrid modelling where channel opening and closing are stochastic processes while calcium diffusion is more efficiently modelled as a deterministic process [26]. In this example we assume the existence of only one channel which permits the flow of calcium to the cytoplasm when it is in the open state. We use two discrete places, *open* and *close*, to model the channel states, open and close, respectively. Likewise, the two stochastic transitions, *ch_open* and *ch_close*, model the processes of opening and closing the channel, respectively. When the channel is in the open state, the calcium can flow from the endoplasmic reticulum (not represented in this example) and enter the cytoplasm, which is represented by the continuous place *Ca*. The continuous transition *Ca_inflow* models this process. Finally, calcium can return back to the endoplasmic reticulum through a process called pump [26]. We model this process using the continuous transition *C* *a*_*p* *u* *m* *p*. Figure 1 b depicts the dynamics of channel opening and closing, while Fig. 1 c provides the corresponding calcium concentrations. For the purpose of this example we have set the parameter values so that we can demonstrate the basic idea which has no immediate biological relevance. The corresponding Snoopy file is given in the Additional file 1: S1.

Beyond these extensions, Petri nets have also been extended in a parameterised way. Such an extension is called coloured Petri nets (${\mathcal {PN^{C}}}$) [27, 28]. In a ${\mathcal {PN^{C}}}$, a group of similar components can be abstracted into one component (similar to a variable), each of which is defined as and thus distinguished by a colour (a specific value of the variable). In a ${\mathcal {PN^{C}}}$, one or more colour sets have to be defined, and a colour set is assigned to each place. The tokens on a place are now distinguishable by colours. A guard, which is a Boolean expression, is assigned to each transition. For enabling a coloured transition, we not only check if the preplaces of the transition have sufficient and appropriate tokens, which is similar to what is done in standard Petri nets, but also have to evaluate the guard, which has to yield true. Each uncoloured Petri net class can have a coloured counterpart. Thus by combining the parameterised modelling capability of ${\mathcal {PN^{C}}}$ and the hybrid representation capability of ${\mathcal {GHPN}}$, we obtain coloured hybrid Petri nets (${\mathcal {GHPN^{C}}}$) [15], which can conveniently model a system having both multiple spatial and temporal scales. In what follows we refer to ${\mathcal {GHPN}}$ and ${\mathcal {GHPN^{C}}}$ simply by ${\mathcal {HPN}}$ and ${\mathcal {HPN^{C}}}$, respectively, unless explicitly stated otherwise.

**Fig. 2:**
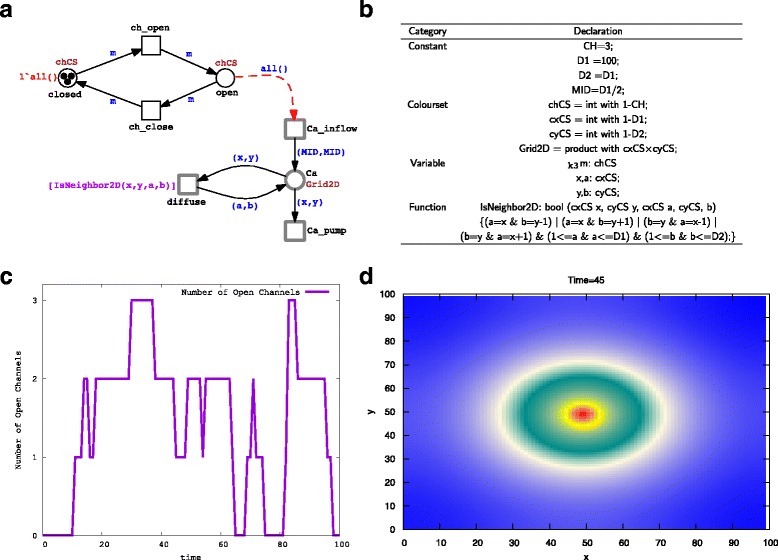
A simple example representing the operation of ${\mathcal {HPN^{C}}}$: (**a**) the ${\mathcal {HPN^{C}}}$ representation of spatial calcium dynamics, (**b**) the corresponding colour definitions, (**c**) a time course representing the number of channels in the open state, and (**d**) a matrix plot representing the calcium diffusion. In this model, we use three channels arranged in one cluster. The colour set *chCS* provides the number of channels (in this case three). The variable *m* is used in combination with the transition *ch_open* to model the transition of a certain channel. The coloured place *open* provides the total number of channels in the open state, which is used as a rate for the continuous transition *Ca_inflow*. The calcium is represented by the coloured place *Ca*, which when unfolded gives a number of places equal to the colours in the colour set *Grid2D* (in this case *Grid2D* is a two dimensional coloured set, each dimension being 100). Calcium diffusion is modelled via the continuous transition *diffuse*. When the continuous transition *Ca_inflow* fires, it adds calcium to the position of the cluster in the grid (here in the middle of the grid (50,50)). The calcium outflow is modelled by the continuous transition called *Ca_pump*

To demonstrate the basic idea of ${\mathcal {HPN^{C}}}$, we extend the example presented in Fig. 1 to include more than one channel arranged in one cluster and account for the spatial behaviour of calcium diffusion. This scenario will be much more realistic than the simple one presented in Fig. 1. Figure 2 a shows a simple example of the calcium dynamics modelled as ${\mathcal {HPN^{C}}}$. The corresponding colour declarations are given in Fig. 1 b. Now the coloured discrete place *closed* is parameterised with the coloured set *chCS* which contains the colours from 1 to 3. Therefore it represents the state of three channels when they are closed, so does the coloured discrete place *opened*. In this coloured model version the two transitions *ch_open* and *ch_close* are bound with each colour in the colour set *chCS*. That is each transition has three different instances corresponding to the number of channels. Moreover, the calcium concentration is modelled by the continuous place *Ca*, which is associated with the colour set *G* *r* *i* *d*2*D* to represent a two-dimensional grid of 100×100 cells (colours). Each of them represents a spatial calcium location. The calcium flow is modelled by the continuous transition *C* *a*_*i* *n* *f* *l* *o* *w* which adds calcium to the cluster location (here assumed to be in the middle of the grid: (50,50). The rate of the transition *C* *a*_*i* *n* *f* *l* *o* *w* is proportional to the total number of open channels in the cluster (see [26] for more details). The calcium diffusion is modelled by the continuous transition *diffuse* which diffuses the calcium to the four neighbouring cells of a calcium position. The calcium pump is done via the coloured continuous transition *C* *a*_*p* *u* *m* *p* which positions a transition at each location in the grid. Figure 2 c depicts the total number of channels in the open state, while Fig. 2 d shows the calcium diffusion in the two dimension coordinates. In this example we can easily carry out different experiments by reconfiguring the model parameters. For instance, the number of channels in the cluster can be increased by just increasing the number of colours in the coloured set *chCS*. Similarly, the grid coordinates can be adjusted by changing the colour set *G* *r* *i* *d*2*D*. The Snoopy file for this introductory example is given in the Additional file 2: S2. A detailed discussion of simulating this coloured model is provided in the “Implementation” section.

## Implementation

In this section we briefly describe the implementation of Snoopy’s hybrid simulator by considering the architecture, available algorithms, export and import, and the deployed external libraries.

### Architecture

**Fig. 3:**
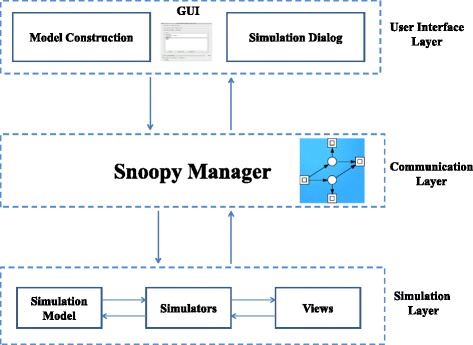
Architecture of Snoopy’s hybrid simulator. The different components of the architecture can be divided into three layers: user interface, communication, and simulation layer. The user interface is the user access point to construct and execute hybrid models. The simulation layer comprises the simulator as well as the simulation version of the constructed model definition. The communication layer connects the simulation component with the user interface

**Fig. 4:**
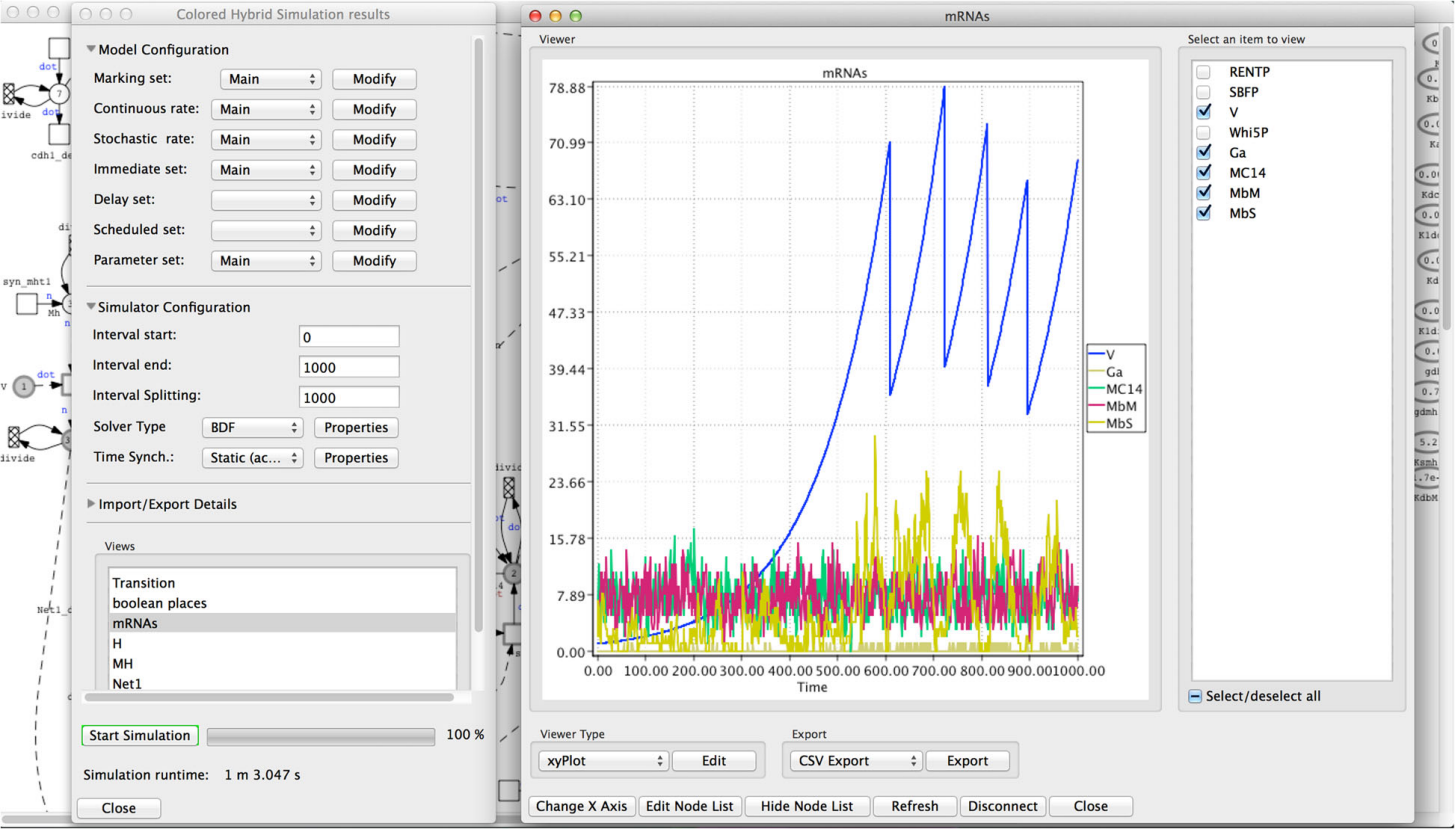
Screenshot of Snoopy’s graphical user interface. The simulation window is divided into two parts: configuration and viewer subwindows. The configuration window (*left*) permits to configure and control the simulation while the viewer window (*right*) is used to display the simulation results. Multiple viewer windows can be opened simultaneously to show the results from different perspectives. The viewer curves can be exported into CSV file for further analysis of the simulation results

Figure 3 presents the architecture of Snoopy’s hybrid simulator. This architecture consists of three components: the user interface, which comprises the model editor and the simulation dialog; the simulator, which implements the simulation algorithms as well as storing the currently running models and the corresponding result views; and the Snoopy manager, which connects the user interface with the simulation module. Snoopy’s hybrid simulator deploys a simple graphical user interface to permit a rapid configuration of the core simulation procedure. Figure 4 depicts the user interface; in the following, we discuss each of these components.

#### Model editor

The model editor permits the graphical construction of hybrid models using (coloured) hybrid Petri net notations defined in [11]. Reactions are represented by transitions, while species are denoted by places. More information about hybrid Petri net notations can be found in [11, 19, 29] as well as in Snoopy’s hybrid simulator user manual [21]. In addition to specifying all reactions, the model editor provides other features to configure the model parameters as well as the initial state. The model editor is applied as a pre-step before executing the simulation.

#### Simulation dialog

The simulation dialog is the user tool to run and manage the simulation. Through it the simulation experiment can be configured and then executed. Moreover, the simulation dialog provides access to the simulation algorithms that are implemented in Snoopy’s hybrid simulator. Once a model has been constructed, a user can access the simulation dialog through Snoopy’s menu bar. The simulation dialog consists of four parts: model configuration, simulator configuration, import/export, and simulation state (compare Fig. 4).

The model configuration section permits to adjust model settings including initial state, reaction rates and other similar parameters. The simulation configuration section deals with specifying the simulation options including the start and end time point of the simulation, the type of the ODE solver, and the hybrid synchronisation method. The import/export section allows users to configure how Snoopy performs any export or import of simulation results. Finally, the simulation state section serves to start and stop the simulation as well as to monitor the simulation state.

The simulation results can be examined using views. Different result views can be defined to explore the simulation output from different perspectives. Each view has its own window to display the results using a dedicated result viewer. Result viewers permit to render the final data using different plotting techniques such as xy-plot. Finally, view curves can be exported into comma separated files (CSV) for further processing.

#### Simulation model

After a model is constructed, e.g. using the model editor, it can be sent to the simulator for execution. The simulation module takes a copy of the hybrid Petri net model, but ignoring the layout information. Usually, the model is a collection of species, reactions, stoichiometries as well as associated data such as kinetic rates and kinetic rate constants. Nevertheless, this information is mapped in terms of Petri net components. When the simulation model is partitioned into deterministic and stochastic parts, reactions and species are assigned to either of the two regimes. Unlike other implementations (e.g., in [10]), we consider only one version of the species: either discrete or continuous. Later, if a transformation is required (e.g., from number of molecules to concentration, or vice versa), this can be easily done at the position where such a conversion is required (e.g., in the rate equation). Similarly, when considering the simulation output, such a transformation can also be easily applied. However, if a species is manipulated by both a deterministic and a stochastic reaction, it needs to be represented as a continuous species.

#### Simulator

The simulation algorithms are implemented as a stand-alone, but built-in simulation library. The simulator module reads the model to be simulated and carries out the execution. A number of algorithms, which will be discussed in the next section, are available to execute a hybrid model. Please note, although the core simulator is implemented as a stand-alone library, it can currently not be used as a stand-alone application. However, we are working on this to achieve a stand-alone application (see the “Future improvements” section).

#### Views

Views are associated with models. Each view is defined over a set of places or transitions of which the dynamic behaviour shall be displayed when the simulation starts. These place/transition sets can be specified by a regular expression. Multiple views can be defined for the same model. A view is also associated with a viewer that displays the selected information. Views can be manipulated or removed after they were initially added to a model.

#### Snoopy manager

The communication between the user interface and the simulator is done via the Snoopy manager. The Snoopy manager acts as an intermediate agent that sends the GUI command to the simulator and gets the result back to visualise or export them to the chosen file format. As Snoopy is a stand-alone application, the communication between the user interface and the simulation module is done internally and not through a physical communication channel.

### Available algorithms

Snoopy’s hybrid simulator encompasses a set of simulation algorithms that together provide a convenient execution of hybrid biological models. The general idea of the hybrid simulation algorithms implemented in Snoopy is as follow. First, the synchronisation module (the hybrid algorithm) prepares the jump equation (see below). Afterwards, the ODE solver numerically integrates the system of ODEs due to the deterministic part until the jump equation is fulfilled. At this point, the synchronisation module switches the control to the stochastic module to select and fire a stochastic reaction. The exact time point of the stochastic event is determined by the jump equation. In what follows, we outline each of these algorithms.

#### Haseltine and Rawlings algorithm

This is the realisation of the hybrid simulation idea proposed by Haseltine and Rawlings in [9]. According to this method, a system of ODEs is numerically solved until a stochastic event is to occur. The exact occurrence time of the stochastic event is captured through (1).

1 $$ \int_{t}^{t+\tau}{\sum\limits_{j=0}^{N}{a_{j}^{s}}(\mathbf{x})dt}=-log(r) \,,   $$

where **x** is the state vector of the model at time *t*, *τ* is the firing time of the next slow reaction, *r* is a random number uniformly distributed in the interval *U*(0,1), $a_{j}^{s}(\mathbf {x})$ is the propensity of the *j* ^*th*^ slow reaction, and *N* is the number of stochastic reactions.

In (1) we aim to determine the value of *τ*. This is achieved by first generating a random number from *U*(0,1) and then integrating the propensity equations of all slow reactions together with the system of ODEs due to the deterministic part from the current simulation time *t* until (1) is satisfied. At this point we know that there is a stochastic event which needs to be fired.

Although this method is very accurate, it requires considerable time to switch from stochastic to deterministic simulation [13]. The performance of this method drops rapidly as soon as the number of stochastic events increases. Thus it is suitable only for simple models where the number of potential stochastic events is limited. Moreover, it can produce better results with ODE solvers that do not collect and use history information to advance the numerical integration time.

#### Accelerated Hybrid Simulation

To overcome the limitation of the Haseltine and Rawlings method, we follow an accelerated approach introduced in [13]. The accelerated algorithm takes advantage of the model structure to boost the overall simulation performance. According to this method, stochastic reactions are classified into two groups: dependent and independent. Dependent reactions affect the system state of the ODE solvers when they occur, while independent reactions have no effects. Therefore, the ODE solver is reinitialised only when a reaction in the dependent group is fired. Thus, the simulation performance becomes better than for the Haseltine and Rawlings method, particularly for bigger models. For instance, in [13] we compared the performance of the Haseltine & Rawlings method and the accelerated approach using three models. We found that there is a notable performance improvement for all three case studies and for certain models; the latter approach is ten times faster than the former one. This save in runtime is mainly due to the reduction of the number of times where the ODE solver is reinitialised. In order to achieve the better performance, the accelerated method approximates the exact capture of the stochastic event occurrence time given by (1) by another equation given in (2).

2 $$ {\sum\limits_{j=0}^{N}{a_{j}^{s}}(\mathbf{x})}\cdot \Delta \tau =-log(r)\,,   $$

where *Δ* *τ* is the time difference between the occurrence time of the previous event and the current event. Eqs. 1 or (2) has to be satisfied during the integration, when the Haseltine and Rawlings or accelerated method is used, respectively, until the ODE solver stops and returns the control back to the stochastic regime. Please note that although our approach mainly intends to reduce the reinitialisation of ODE solvers employing history information to advance the simulation time (e.g., multi-step ODE solver; see below), it can also be used with single step solvers (e.g., Runge-Kutta) to reduce the frequent recalculation of the step size after each firing of a discrete event.

#### Improved hybrid rejection-based stochastic simulation

The numerical integration of (1) as well as its approximation in (2) are computationally expensive to be satisfied. Therefore, in [12] a new hybrid simulation method was proposed based on the rejection-based stochastic simulation algorithm (RSSA) introduced in [30] which avoids the calculation of (1) and (2). The RSSA algorithm defines lower and upper bounds of the reaction propensities to minimise the propensity updates. The propensity lower and upper bounds are calculated based on a lower and upper bound of the system state values called fluctuation interval. Propensities are updated only when one or more of the system state entries move completely outside the defined fluctuation interval. The Hybrid Rejection-based Stochastic Simulation Algorithm (HRSSA) exploits this opportunity by switching from the deterministic to the stochastic regime only when the ODE solver reaches the time of a stochastic event or when any of the system state entries is outside the fluctuation interval. In the former case, the discrete regime does not affect the continuous one, while in the latter case the deterministic regime changes the state of the discrete species during the numerical integration. We apply an improved implementation of this method which combines the accelerated and hybrid rejection-based methods. Currently, the improved hybrid rejection stochastic simulation method is tested as the best hybrid algorithm implemented in our tool in terms of performance (compare Table 2). In [12], the performance of the HRSSA algorithm has been compared with state of the art hybrid simulation algorithms using five benchmark models. It turned out that the HRSSA outperforms all competing algorithms.

#### Dynamic hybrid simulation

The previously discussed simulation approaches are based on static partitioning. Static partitioning adopts a predefined classification of the model reactions into stochastic and deterministic ones. The partitioning itself is usually performed by the user and exploited afterwards by the simulator during the whole simulation process. This approach is constructive for many applications with a clear cut between reactions which have to be simulated stochastically and those which should be simulated deterministically. For instance, in [31] and in many other similar publications that study cell fate, reactions related to the cell nucleus are considered as stochastic, while those happening inside the cytoplasm are considered as deterministic. However, such a clear cut is not always possible to be achieved for all models during the whole simulation period. Reactions can change their state from slow to fast and vice versa during the simulation. For example, in oscillating biological systems, reaction rates also oscillate with respect to time from fast to slow and the other way around. In this case, dynamic partitioning, where reactions are partitioned repeatedly during the simulation, can play a role in speeding up the whole simulation procedure. Our implementation of the dynamic hybrid simulation is based on the improved hybrid rejection method. Using this approach, reactions are repartitioned as soon as any of the state vector entries leaves the fluctuation interval. This will indeed eliminate the need for frequent checks of whether the set of reactions requires repartitioning.

#### Pure stochastic and pure deterministic simulation

To improve the comfort when simulating biological models with Snoopy’s hybrid simulator, the user has the option to perform a pure stochastic or a pure deterministic simulation of a hybrid model. The direct method [3] is applied to implement the stochastic simulation, while the SUNDIAL CVODE [32] is used to carry out the deterministic simulation. This is a worthwhile feature during the experimentation phase to compare the hybrid results with the pure stochastic and pure deterministic ones. Using this feature, Snoopy’s hybrid simulator ignores any reaction partitioning specified by the user and reads all model reactions as stochastic or deterministic ones, depending on the selected simulation algorithm.

#### Parallel multi-run simulation

Similar to stochastic simulation, hybrid simulation of biological models might require the execution of multiple runs to calculate average statistics. Snoopy’s hybrid simulator permits the concurrent execution of different runs to take advantage of the existence of multiple cores in the user’s machine.

### Simulation of coloured models

A coloured model (such as ${\mathcal {HPN^{C}}}$) with finite colour sets can be automatically unfolded to an uncoloured model (such as ${\mathcal {HPN}}$). See [33] for one of the unfolding algorithms deployed in Snoopy. Thus, the simulation of an ${\mathcal {HPN^{C}}}$ model is done on the automatically unfolded ${\mathcal {HPN}}$ model. When the user starts the simulation of an ${\mathcal {HPN^{C}}}$ model, an unfolding dialogue will be triggered, where the user can select an appropriate unfolding method to perform the unfolding. Afterwards, the simulation methods discussed in this section can be used to execute the unfolded model.

To better explain this idea, we consider the ${\mathcal {HPN^{C}}}$ model presented in Fig. 2. First, the discrete subnet, consisting of the two places: *closed* and *open* as well as the two transitions: *c* *h*_*o* *p* *e* *n* and *c* *h*_*c* *l* *o* *s* *e*, is unfolded into three identical subnets (because the colour set *chCS* consists of three colours). In other modelling scenarios where the unfolded subnets are not identical we can make use of transition guards to imply the required constraints. Similarly, the continuous subnet, consisting of the place *Ca* and the two transitions *C* *a*_*p* *u* *m* *p* and *diffuse* is unfolded into 10,000 identical subsets (because the colour set *G* *r* *i* *d*2*D* consists of 100×100 colours). However, because the transition *diffuse* has a guard expression, only transitions for the colours satisfying this Boolean expression are added. The transition *C* *a*_*i* *n* *f* *l* *o* *w* will have only one copy in the unfolded net because the input and output arcs contain a constant expression. Nevertheless, this procedure does not need to be implemented iteratively as we do in this small example. Instead, it can be viewed as a constraint satisfaction problem (CSP) which can be solved by a dedicated CSP solver (e.g. [34]).

### Export and import

Snoopy supports the import/export of Petri net models from/to other tools and formats. First, Snoopy imports and exports the (C)ANDL format (cf. [21]) which is a human readable file format used by other software tools (e.g., Marcie [35] and Charlie [36]) which can be employed for a formal analysis of Petri net models (e.g., structure analysis, model checking, etc.). Moreover, Snoopy reads and writes SBML files [37] according to SBML level 2 version 4 by using libSBML [38]. However, we support only a subset of SBML elements that is compatible to our net classes; specifically we do not support any kind of rules or events. Snoopy passes all tests of the SBML Test Suite comprising supported elements. However, the partitioning of hybrid models is lost when exporting to SBML, because SBML has no support for hybrid models yet. The user has to decide whether the exported model has to be treated stochastically or continuously. Furthermore, a coloured model is exported to SBML by first unfolding it into the low level representation and then performing the export.

### Implementation language and external libraries

Snoopy’s Hybrid Simulator has been implemented using standard C++. As a component of Snoopy, it adopts wxWidget [39] to implement the graphical user interface under different operating systems. Moreover, the stochastic and deterministic simulation components are implemented in a modular way such that different algorithms can be easily exchanged to execute the stochastic and deterministic regimes. Snoopy’s hybrid simulator adopts internally an external library, SUNDIAL CVODE [32], to solve a system of ODEs. The ODE library provides two main algorithms: one for stiff and one for non-stiff ODEs. Additional ODE solver modules can easily be added in future releases. We also make use of the C++ library Boost [40] to carry out routine tasks such as input parsing and multithreading support.

## Results

### Snoopy’s hybrid simulator provides a graphical and convenient way to construct hybrid models

Before using Snoopy’s hybrid simulator, a model needs to be constructed by specifying reactions, species, stoichiometries, kinetic rates, etc. In Snoopy a model is usually constructed using Petri net notations. However, existing models can also be imported from other formats including the well known SBML. In what follows, we present two methods that permit the construction of hybrid models in Snoopy.

#### Simple models

For simple models which involve a limited number of reactions and species (e.g., both less than 100), we use ${\mathcal {HPN}}$ to construct them. Snoopy’s hybrid simulator supports two types of places, five types of transitions and six arc types to facilitate the convenient modelling of hybrid biological systems. A complete description of these elements is provided in the user manual [21]. Unlike other hybrid Petri net tools and similar to the semantics discussed in [25], we apply the bio-semantics to execute the continuous part of the ${\mathcal {HPN}}$ (see also the “Background” section), which is more efficient than the adaptive semantics when simulating biological models.

#### Coloured models

For large-scale biological systems, the corresponding ${\mathcal {HPN}}$ models become difficult to manage. In this case, we may use ${\mathcal {HPN^{C}}}$ (see the “Background” section) for model construction. These models may exhibit many repeated components as well as spatial behaviour (see Fig. 2 for an example). Colours have been successfully deployed to model many real biological applications (for examples see [28, 41, 42]).

### Snoopy’s hybrid simulator provides an efficient way to execute hybrid models

Once a model has been created, it can be simulated using one of the algorithms discussed in the previous section. The simulation dialog has been designed to be intuitive with many options to configure the simulation. Furthermore, the resulting time course data can either be viewed inside Snoopy or exported for further processing. In the following we summarise the required steps to execute a hybrid model. A more detailed discussion can be found in [21].

1. **Configure the constructed model:** Before running the simulation, you may need to adjust the model setting (see the model configuration section in Fig. 2), which includes choosing the initial state and/or the kinetic rate constants.
2. **Configure the chosen simulator:** To run a hybrid simulator you have to select an appropriate synchronisation algorithm, which is one of the discussed hybrid simulation methods, as well as the type of the ODE solver. Moreover, depending on the specific model, a user might need to adjust the options of the ODE solver. For many hybrid models, the default settings can be kept. However, Snoopy’s hybrid simulator offers a wide range of other options for complex hybrid models that require special treatment (see the simulation configuration section in Fig. 2).
3. **Run the simulation and explore the results:** After the model and the simulator are configured, the simulation can be started. You may need to create a new result view to explore the simulation output.
4. **Export the simulation results:** As a final step, the resulting data can be exported to a CSV file for further post-processing.

### Example of using Snoopy’s hybrid simulator

To demonstrate how Snoopy’s hybrid simulator can be used to deal with hybrid biological models, we include a sample application and show how this model can be constructed using ${\mathcal {HPN}}$ notations and then executed via a hybrid simulation algorithm.

When studying cell fate behaviour, where a cell decides either to undergo cell cycle arrest or commit apoptosis [43–45] in response to DNA damage, a model describing this phenomenon can be clearly divided into two parts: one with species exhibiting low number of molecules and the other one involving species with high number of molecules. In such cases, it may not be feasible to apply stochastic simulation due to the huge number of stochastic events.

**Fig. 5:**
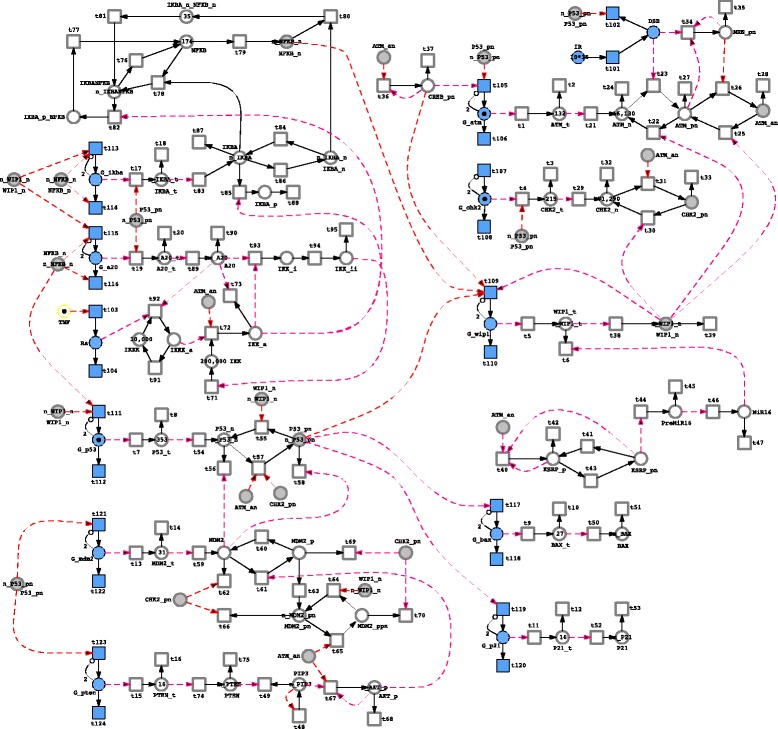
Implementing the ATM/P53/NF- *κ*B pathway model from [31] using Snoopy’s hybrid simulator. *Circles* (places) represent species, *squares* (transitions) represent reactions, and arcs denote connections between the two node types. More information about these notations can be found in [11, 21]. *Coloured circles* represent discrete species, while the uncoloured ones represent continuous species. Similarly, *coloured squares* represent stochastic reactions, while uncoloured ones denote continuous reactions. *Solid black arcs* represent connections that consume molecules when the corresponding reaction fires, while *dashed coloured* ones just permit the use of substrates for defining reaction rates. *Grey* nodes are logical places that are repeated to simplify the network layout. Please note, inhibitor arcs (arc with *small circles*) enforce the number of genes to be at most two [31]. The complete Snoopy file is provided in the Additional file 2: S2.

We deploy a recent model from [31] as a sample use case for illustrating Snoopy’s hybrid simulator. This model permits to investigate the importance of various DDR (DNA Damage Response) elements after DNA damage induction during cell fate determination. More specifically, the model studies the ATM/p53/NF- *κ*B pathway, consisting of four main modules: p53 (a tumor suppressor protein), ATM (ataxia telangiectasia mutated), NF- *κ*B (a nuclear transcriptional factor) and Wip1 (a p53-induced protein phosphatase), and involves three different compartments: nucleus, cytoplasm, and extracellular matrix [31]. The main aim of the model is to explore the connection between these four key proteins and protein phosphates in order to understand cellular response to DNA damage which is important to understand cell fate determination. A key model component is Wip1, which is increased to a level that can block the corresponding cell apoptotic decision when DNA repair is successful [31]. However, the level of Wip1 should not stay high after DNA repair; otherwise the cell will not be sensitive to future damage. The model is divided into the following stochastic and deterministic parts. All the genes such as Wip and ATM are considered as discrete places, and all reactions related to genes (gene expression and degradation) are kept stochastic, while all the other species are considered as continuous places, and all other reactions, except those related to DSB (DNA double-strand breaks) creation and repair, are modelled as deterministic transitions. The output of the model consist of the levels of molecules with respect to time after irradiation and also the cell fate decision. Figure 5 depicts the Snoopy implementation of this model.

**Fig. 6:**
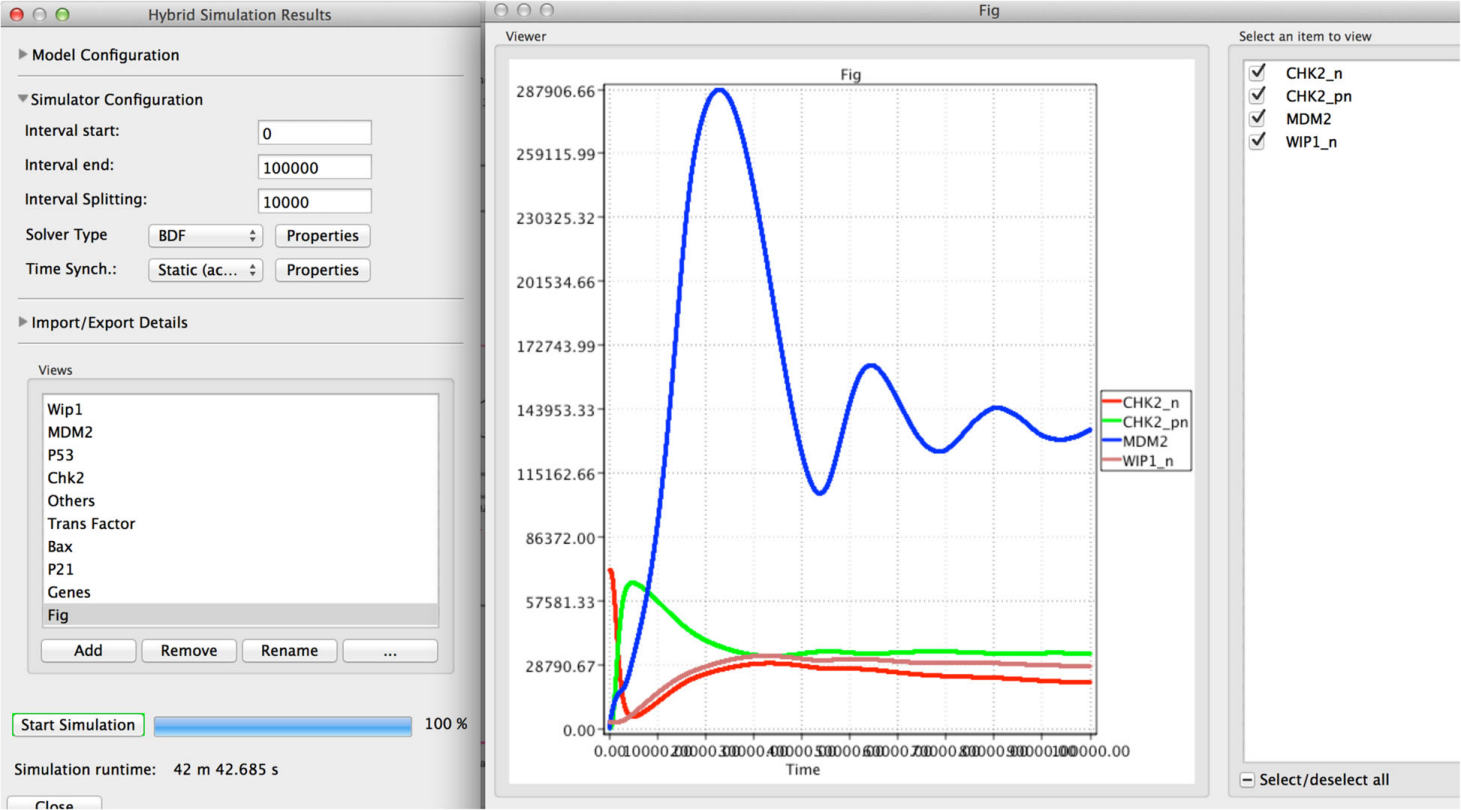
A screenshot of simulating the ATM/P53/NF- *κ*B pathway model via Snoopy’s hybrid simulator. The different curves represent the time course behaviour of four model species. The simulation is done by executing the model given in Fig. 5 for the time period from 0 to 100,000 seconds with the accelerated algorithm

The accelerated hybrid simulation algorithm [13] is chosen to execute this model due to the weak coupling between the two reaction regimes. Figure 6 gives a screenshot of simulating the model using Snoopy’s hybrid simulator which includes the time course behaviour of two versions of the gene Checkpoint kinase 2 (CHK2): inactive (CHK2_n,) and active (CHK2_pn,), in addition to the negative regulator protein of the p53 (denoted by MDM2), and the nuclear version of Wip1 (Wip1_n). The simulator output in Fig. 6 can also be exported to a CSV file for further processing. For example, in this model it may be required to count the number of cells that undergo apoptosis and those which exhibit cell cycle arrest. A threshold of the concentration of species p53, P21 (representing the p21 protein) and Bax (denoting the Bax protein) can be used to extract this information [31]. Such post-processing can be done by help of the exported simulation traces. Moreover, a performance comparison of three simulation algorithms when executing this model is provided in Table 2. The Snoopy file is included in the Additional file 3: S3, while a short description of how to execute the model is given in the Additional file 4: S4.

## Discussion

### Installation

Snoopy’s hybrid simulation is installed as part of Snoopy. The Snoopy installation package can be run just by one click on a local computer with one of the three well-known operating systems. No additional dependencies do exist. In other words, all dependent packages are installed with Snoopy’s main package. A detailed procedure of how to install Snoopy on these platforms is given in its user manual.

### Comparison with other tools

In this section we compare Snoopy’s hybrid simulator with two of the well-known software tools that provide a hybrid simulation module to systems biologists: COPASI [16] and Virtual Cell (VCell) [17]. Table 1 summarises the features of COPASI, Virtual Cell, and Snoopy’s hybrid simulator with respect to the hybrid simulation procedures supported by the three tools.

**Table 1:** Comparison of Snoopy’s hybrid simulator with two other similar tools

Features ∖Tools	Snoopy’s hybrid simulator	COPASI [16]	VCell [17]
Use of graphical notations to specify model reactions	Yes	No	Yes
Use of a parameterised language to manage larger models	Yes	No	No
Support unstiff ODE solvers	Yes	Yes	Yes
Support stiff ODE solvers	Yes	Yes	Yes
Improving simulation performance by analysing the model structure	Yes	No	No
Interplay of stochastic and deterministic modules	Variable	Fixed	Fixed
Exact hybrid simulation	Yes	Yes	No
Platform-independent	Yes	Yes	Yes
Availability	Free	Free	Free

COPASI [16] is a general-purpose software tool for constructing and executing computational biological models. It provides tables and widgets as user interface to specify compartments, reactions, species and other related parameters. It reads and writes models written in SBML. For hybrid simulation, COPASI adopts a version similar to the Haseltine and Rawlings method, which has been independently developed at the same time [1, 16]. However, it deploys a tight coupling of the SSA and a specific ODE solver. To be precise, COPASI offers an hybrid Runge-Kutta/SSA algorithm, combining the classical Runge-Kutta ODE solver with the SSA algorithm, an LSODA/SSA, combining LSODA – a dynamic switching between stiff/nonstiff solvers – with the SSA algorithm, and recently it has been extended to support HybridRK-45. COPASI is platform-independent and is available free of charge.

The Virtual Cell [17] modelling and analysis tool also provides a module to execute hybrid models. Virtual Cell is deployed as a distributed application that can be downloaded free of charge. It uses the BioModel as well as VCell Markup Language to construct cell models. Three hybrid algorithms are supported: Hybrid (Gibson/Euler–Maruyama), Hybrid (Gibson/Milstein), and Hybrid (Adaptive Gibson/ Milstein).

Compared with COPASI and Virtual Cell, Snoopy’s hybrid simulator offers a set of features that can improve the performance as well as the productivity of constructing and executing hybrid biological models. These include: analysing reaction networks to accelerate the simulation, implementing a modular design of the stochastic and deterministic procedures, implementing the state of the art of hybrid simulation algorithms, deploying accurate and efficient hybrid simulation algorithms, and utilising a parameterised language (coloured hybrid Petri nets) to construct large scale biological models.

On the one hand, Snoopy’s hybrid simulator makes use of the structural information of the underlying reaction network to boost the overall simulation performance. For instance, the accelerated hybrid simulation algorithm, presented in [13], increases the performance of some hybrid models by ten times compared to the classical Haseltine and Rawlings method as it has been asserted in [13]. This improvement in the runtime is mainly due to detection of reaction dependencies between the deterministic and stochastic regime. In other words, Snoopy’s hybrid simulator avoids unnecessary re-initialisations of the ODE solver when the system state of the ODE solver is not affected by the firing of the current discrete event.

On the other hand, Snoopy’s hybrid simulator does not assume a fixed combination of the ODE solvers and the SSA algorithms as in COPASI and Virtual Cell. Instead, a user can select the appropriate type of the ODE solver, and the hybrid simulation algorithm acts as a time synchronisation module. Such modular design facilitates the support of new ODE solvers and SSA algorithms in the future with minimal efforts. Moreover, the user can take advantage of this modular design by selecting a different combination of the stochastic solver, ODE solver, and the hybrid time synchronisation procedure. This feature can be beneficial to address the issue that different models may have their own computational demands.

Furthermore, Snoopy’s hybrid simulator implements the state of the art of hybrid simulation algorithms that have a better performance than the classical ones. For example, Snoopy’s hybrid simulator implements the hybrid rejection-based stochastic simulation algorithm which has been recently introduced in [12]; it represents a promising direction to improve hybrid simulation when dynamic and static partitioning strategies are used.

To improve the simulation performance, the previous hybrid simulation algorithm implemented in COPASI does not include time-varying propensities in the slow subsystem [1] (i.e., there is no check for (1) or any similar exact methods, e.g., [12]). Although this approach can improve the simulation performance, it will affect the result accuracy. Recently, a new hybrid module (HybridRK-45,) has been added to COPASI to improve the simulation accuracy and overcome this limitation. On the contrary, Snoopy’s hybrid simulator implements three exact versions of the algorithm in [9]. Moreover, recent advances of the theory of hybrid simulation (e.g,. in [12] and [13]) render it possible to overcome the computational overhead to check (1) or an alternative as (2).

Finally, unlike COPASI and Virtual Cell, Snoopy’s hybrid simulator deploys a special parameterised language, coloured hybrid Petri nets, to deal with larger models which cannot be easily managed using traditional model construction approaches (see Fig. 2 for an example).

Compared with the different simulation approaches discussed in [1], Snoopy’s hybrid simulator mainly supports three hybrid algorithms that consider time-varying propensities. That is the changes in the propensities of slow reactions, while the deterministically simulated reactions are evolving, are exactly captured using (1), (2), or using the approach introduced in [12]. The biochemical reaction networks can either be partitioned by the user (i.e., the net is drawn by the user as stochastic and deterministic subnets), or it can be partitioned online by Snoopy’s hybrid simulator. In the latter case the reaction propensities as well as the number of molecules in the reaction substrates serve as criteria to carry out the partitioning.

### Performance measures

To evaluate the performance of the three main algorithms implemented in Snoopy’s hybrid simulator, we give the runtimes of four case studies as performance measures. The case studies range from simple to complex ones that involve many species and reactions. These include: a T7 phage model [8], a hybrid model of the eukaryotic cell cycle [29] based on the stochastic one in [7], the ATM/p53/NF- *κ*B model [31] which has been discussed in the “Results” section, and the simple hybrid calcium model provided in Fig. 2. The simulation experiments have been conducted on a Mac Pro. with 3 GHz Core i7 processor and 8GB memory.

Table 2 summarises the number of species and reactions as well as the runtime of each example model when they are simulated using each of the three hybrid algorithms. For the T7 model we use the partitioning scheme discussed in [11], while for the eukaryotic cell cycle model we apply the same partitioning as discussed in [29]. For the purpose of performance comparison we provide the number of stochastic events produced by each model. The percentage numbers given in parentheses represent the speed of the accelerated and improved algorithms compared to the Haseltine and Rawlings algorithm, and negative values mean the latter algorithm is faster.

**Table 2:** Performance measures of the three implemented algorithms in Snoopy’s hybrid simulator

Measures ∖Models	Model Information			Runtimes of Simulation Algorithms (sec.)		
	Species (Discrete)	Reactions (Stochastic)	Stochastic Events	Haseltine& Rawlings ^∗^	Accelerated Method	Improved HRSSA
T7 Phage (1,000 runs)	3 (2)	6 (4)	4,238,978	120.2	41.7 (281%)	26.6 (452%)
ATM/p53/NF- *κ*B	62 (14)	119 (24)	3,726	6.3	1.9 (332%)	0.96 (656%)
Cell Cycle Model	26 (14)	51 (20)	762,612	870.4	625.7 (139%)	365.5 (238%)
Calcium Model (Fig. 2)	10,006 (6)	49,607 (6)	29	215.8	220.7 (-1.1%)	190 (1.13%)

^*^This refers to the algorithm version which exactly accounts for Eq. (1)

In the Haseltine and Rawlings algorithm, the runtime required to simulate a model mainly depends on the number of corresponding stochastic events. This fact is illustrated by the four case studies. For instance, although the T7 phage model consists of only six reactions (four of them are simulated stochastically), it takes a considerable runtime compared to the calcium model where ten of thousands of reactions are involved. This is not the case in the accelerated and improved HRSSA algorithms, since not all of the stochastic reactions affect the deterministic solver. For instance, in the ATM/p53/NF- *κ*B model, there is a substantial gain in terms of the runtimes because only very few stochastic events trigger a reinitialisation of the ODE solver (compare Fig. 5). Moreover, the runtime for the calcium model is comparable for all three algorithms because there are only a few stochastic events and the optimisation by the accelerated and improved HRSSA algorithms does not play a role.

Although the number of stochastic events in the cell cycle model is less than those in the T7 phage model, the latter model takes less runtime. The extra runtime is taken by the ODE solver, since the numerical integration of the cell cycle model exhibits more discontinuities, due to the volume division (cf. [7, 29]) than the T7 phage model where only two reactions are simulated deterministically.

The accuracy of the simulation results is the same for the three simulation algorithms since the core idea has not been changed. The accelerated and improved HRSSA approaches avoid the reinitialisation of the deterministic module for stochastic events which do not have an effect on the deterministic solver. This will not influence the simulation accuracy (please see [12, 13] for more details).

### Future improvements

The development of Snoopy and its hybrid simulator is still active and new features and algorithms can be added in the future to further enrich its simulation capabilities. We will continue to investigate how to further improve the performance of the hybrid simulation by exploiting the model structure. Moreover, we intend to support additional ODE solvers and other stochastic simulation algorithms to execute the semantics of different types of models. Currently, the simulation library depends on Snoopy’s graphical user interface for reading a model. As a future extension of this scenario, we intend to create a command line application that reads SBML files or the Petri net file and simulates them directly using the simulation library. We will also continue to incorporate recent hybrid algorithms to Snoopy’s hybrid simulator. The mailing list snoopy@informatik.tu-cottbus.de is dedicated to potential queries and bugs about Snoopy and its components.

## Conclusions

In this paper we have presented Snoopy’s hybrid simulator, a tool to execute hybrid biological models. Snoopy’s hybrid simulator has been developed over the last five years, and reached recently a mature and reliable state. It employs a variety of hybrid simulation algorithms such that it can deal with various types of biological models that are usually encountered in systems biology. In addition to the simulation capabilities, the model can take advantage of the graphical representation via hybrid Petri nets notations when it is constructed and simulated via Snoopy’s hybrid simulator.

## Additional files

Additional file 1An example ${\mathcal {HPN}}$model. A Snoopy file implementing the calcium dynamics using ${\mathcal {HPN}}$ notations. (HPN 56 kb)

Additional file 2An example ${\mathcal {HPN^{C}}}$model. A Snoopy file implementing the calcium spatial dynamics using ${\mathcal {HPN^{C}}}$ notations. (COLHPN 114 kb)

Additional file 3 the ATM/p53/NF- *κ*B HPN model. A Snoopy file implementing the ATM/p53/NF- *κ*B. (HPN 1193 kb)

Additional file 4 Description of the ATM/p53/NF-kB HPN model. A short description of how to open and simulate the Snoopy file of the ATM/p53/NF- *κ*B HPN model. (PDF 1248 kb)

## Abbreviations

DSB: DNA double-strand breaks
${\mathcal {GHPN}}$: Generalised hybrid Petri nets
${\mathcal {HPN}}$: Hybrid Petri nets
${\mathcal {HPN^{C}}}$: Coloured hybrid Petri nets
ODE: Ordinary differential equation
SBML: Systems biology markup language
SSA: Stochastic simulation algorithm
