# Supplementary material for: Snoopy’s hybrid simulator: a tool to construct and simulate hybrid biological models
Source: BMC Syst Biol. 2017 Jul 28;11:71. doi: 10.1186/s12918-017-0449-6 (PMC5534078; doi:10.1186/s12918-017-0449-6)
Supplement: Supplementary file 4 — Description of the ATM/p53/NF-kB HPN model. A short description of how to open and simulate the Snoopy file of the ATM/p53/NF- κB HPN model. (PDF 1248 kb) [file 12918_2017_449_MOESM4_ESM.pdf]

# S4: Executing ATM/P53/NF-kB Pathway Model using Snoopy Hybrid Simulator

Mostafa Herajy[1], Fei Liu,[2] Christian Rohr[3], and Monika Heiner[3]

[1] Department of Mathematics and Computer Science, Faculty of Science, Port Said University, 42521 - Port Said, Egypt
[2] Control and Simulation Center, Harbin Institute of Technology, Postbox 3006, 150080, Harbin, China
[3] Computer Science Institute, Brandenburg University of Technology Postbox 10 13 44, 03013 Cottbus, Germany
`http://www-dssz.informatik.tu-cottbus.de`

In this short guide, we present a summary of how to execute the example model presented in the main paper and included in the supplementary material S2. For more information about Snoopy hybrid simulator and its different options, please conduct the link:

`http://www-dssz.informatik.tu-cottbus.de/DSSZ/Software/FAQ`

as well as the user manual of Snoopy's hybrid simulator. To execute a hybrid model in Snoopy, please follow these steps:

1. **Download and install Snoopy**
   Snoopy can be downloaded from:
   `http://www-dssz.informatik.tu-cottbus.de/DSSZ/Software/Snoopy`.
   Snoopy is a platform-independent. Therefore, you can download the suitable installation package depending on your operating system. Moreover, you can easily install Snoopy by just one mouse click.
2. **Open the HPN file called S2.sphybrid**
   Once Snoopy is installed on your machine, you can use it to construct and simulate Petri net models. To open an already constructed Petri nets, follow these steps:
   (a) Open Snoopy
   (b) From the *File* menu, select *Open*.
   (c) Locate the HPN model in your file system.
   (d) Click open.

   Snoopy will open the file as shown in Figure 1
3. **Opening the simulation dialog**
   To simulate a Snoopy file, you will need to open the simulation dialog. To open the simulation dialog, select from the *View* menu *Start Simulation-Mode*. You can also press *F6*. The simulation dialog will be open as in Figure 2.

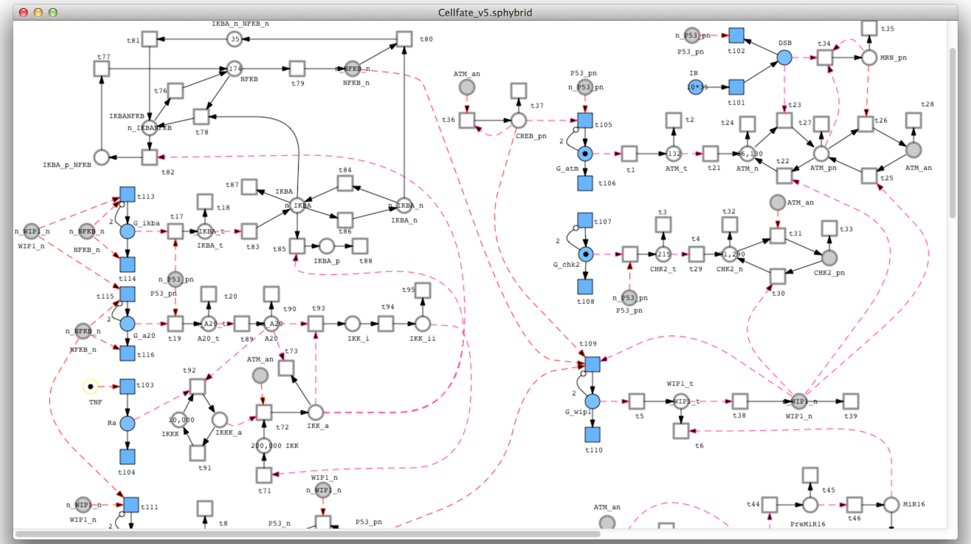


**Fig. 1.** Opening Snoopy under MacOSx.

4. **Configure the simulation**
   There are just few parameters that you might need to adjust before starting the simulation. These include:
   (a) *Interval Start:* Specifies the start time point where Snoopy will begin to record the output. In all cases the simulation will begin at time zero, while output recording will begin at time point specified by *Interval Start:*.
   (b) *Interval End:* The time point where the simulation will stop.
   (c) *Interval Splitting:* The total number of output points that are recorded by the simulator.
   (d) *Solver type* the type of the ODE solver used.
   (e) *Time Synch:* The type of the hybrid simulation algorithm.

   You can adjust these parameters by clicking of *Simulator Configuration* on the simulation dialog. For our model, we have selected the following values of the parameters:
   - *Interval Start:* 0
   - *Interval End:* 100,000
   - *Interval Splitting:* 10,000
   - *Solver type:* BDF
   - *Time Synch:* Static (Accelerated)

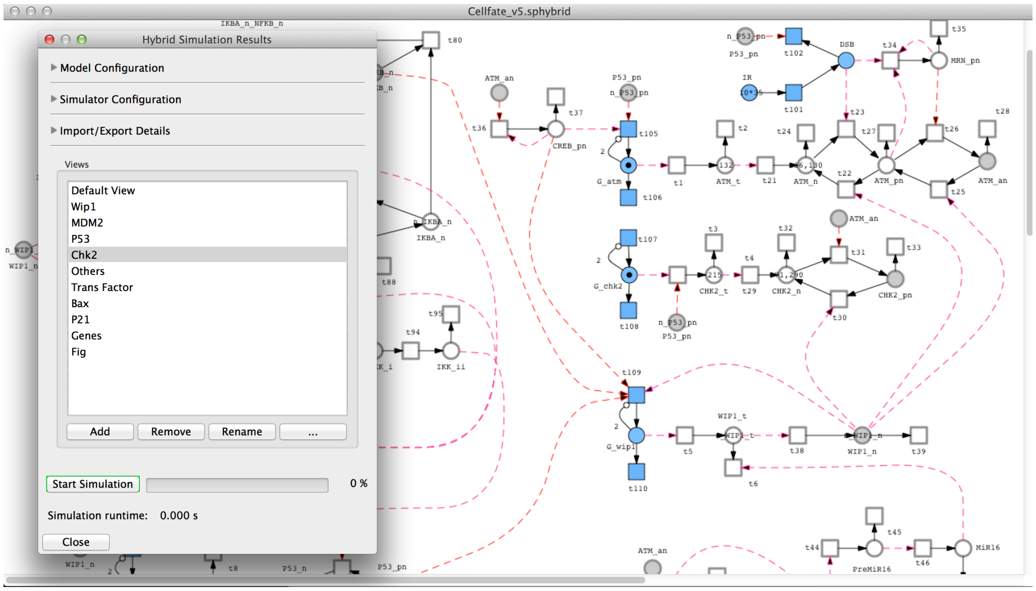


**Fig. 2.** Opening a view in Snoopy.

5. **Execute the simulation**
   To Start the simulation please hit the *Start Simulation* button.
6. **Explore the simulation result**

   Snoopy organises the simulation result in terms of views. Each view can contain a set of places (or transition rates) to show their result. For our model, there are a number of views that are already defined. To explore the curve of the place *Chk2*, select the view called *Chk2* and press *show selected views*.
7. **Export the model result**
   The simulation output can be exported as an CSV format for further manipulation. To export the output curves as CSV do the following steps:
   (a) In the currently open view, press export.
   (b) select the file name and the separator (for gnuplot plot we select "Tab" as a separator).
   (c) Press *exprort*.
